# Identification and Expression Analysis of Long Non-coding RNA in Large Yellow Croaker (*Larimichthys crocea*) in Response to *Cryptocaryon irritans* Infection

**DOI:** 10.3389/fgene.2020.590475

**Published:** 2020-11-12

**Authors:** Xinyi Zhang, Tao Zhou, Baohua Chen, Huaqiang Bai, Yulin Bai, Ji Zhao, Fei Pu, Yidi Wu, Lin Chen, Yue Shi, Qiaozhen Ke, Weiqiang Zheng, Jia Chen, Peng Xu

**Affiliations:** ^1^State Key Laboratory of Marine Environmental Science, College of Ocean and Earth Sciences, Xiamen University, Xiamen, China; ^2^State Key Laboratory of Large Yellow Croaker Breeding, Ningde Fufa Fisheries Company Limited, Ningde, China; ^3^Fujian Key Laboratory of Genetics and Breeding of Marine Organisms, College of Ocean and Earth Sciences, Xiamen University, Xiamen, China; ^4^Xiamen Key Laboratory of Urban Sea Ecological Conservation and Restoration (USER), Xiamen University, Xiamen, China

**Keywords:** *Larimichthys crocea*, *Cryptocaryon irritans*, lncRNAs, infection, immune response

## Abstract

Large-scale transcription studies have revealed numerous lncRNAs (long non-coding RNAs). lncRNAs have been proposed to participate in the regulation of a diverse range of biological processes, including transcriptional regulation. Although lncRNAs have attracted increasing attention, the studies in large yellow croaker (*Larimichthys crocea*) are still rare, and they lack systematic analysis. In this study, 101 RNA-seq datasets varied in ages, sexes, and tissues were retrieved from the NCBI database to generate a comprehensive catalog of large yellow croaker transcriptome database. A set of 14,599 high-confidence lncRNAs from 13,673 loci were identified and characterized. Furthermore, RNA-seq datasets obtained from the infection of *C. irritans* were employed to investigate the differential expression pattern of lncRNAs and analyze potential biological functions. A total of 77 differentially expressed lncRNAs targeting to 567 protein-coding genes were identified by using expression analysis. Several immune genes, including TLR5, CD2AP, and MMP9, were highlighted. With GO enrichment and KEGG pathway analysis, the immune-related terms or pathways were enriched. This study created a comprehensive dataset of lncRNAs for large yellow croaker, which would be helpful for the researches of functional roles of lncRNAs in large yellow croaker.

## Introduction

With the rapid development of high-throughput sequencing technology, the genome-wide identification of RNAs has been performed in a large number of species and helped to discover numerous non-coding RNA genes (Kung et al., 2013; Iyer et al., 2015; Rinn and Chang, 2012). Established databases like NONCODE^1^ and GENCODE^2^ have annotated different classes of non-coding RNAs including long non-coding RNAs (lncRNAs), tRNA, rRNA, and microRNA. lncRNA is a class of non-coding transcripts with length over 200 nt. lncRNAs can affect expressions of local genes and remote genes in *cis/trans* ways as decoys, scaffolds, guides, and enhancers (Kung et al., 2013). Xist is a well-studied lncRNA and an ideal example to demonstrate the regulating function. Xist spreads across one X chromosome and initiates a series of events, including the deposition of repressive chromatin marks leading to expression silencing of almost the entire chromosome in female mammals (Chen et al., 2016; Carmona et al., 2018). lncRNAs also play important roles in the epigenetic process, cell cycle regulation, and cell differentiation (Hung et al., 2011; Lee, 2012; Fatica and Bozzoni, 2014; Kopp and Mendell, 2018). Because of the various functions, non-coding transcriptome profiling analysis has been performed in multiple species, such as mice (Josset et al., 2014), chicken (You et al., 2019), goat (Liu et al., 2018), sheep (Bakhtiarizadeh and Salami, 2019), silkworm (*Bombyx mori*) (Wu et al., 2016), and Pacific Oyster (*Crassostrea gigas*) (Sun and Feng, 2018). In teleost, similar works have been performed in red cusk-eel (*Genypterus chilensis*) (Dettleff et al., 2020), common carp (*Cyprinus carpio*) (Song et al., 2019), tilapia (Li et al., 2018), rainbow trout (*Oncorhynchus mykiss*) (Ali et al., 2018), and zebrafish (Pauli et al., 2012).

The roles of lncRNA in the immune system have attracted considerable attention (Schmitt and Chang, 2016; Fan et al., 2018). In human, mouse, and chicken, detailed function roles for specific lncRNAs in immunity have been elucidated like lincRNA-Cox, HOTTIP, H19, lincRNA-EPS, and lncRNA-CD244 (Carpenter et al., 2013; Chen et al., 2017). In teleost, studies have also been reported about profiling of lncRNAs under different immune challenges, such as salmon, trout, zebrafish, and large yellow croaker (Jiang et al., 2016; Valenzuela-Munoz et al., 2018; Liu et al., 2019; Valenzuela-Munoz et al., 2019).

Large yellow croaker (*Larimichthys crocea*) is the top maricultural fish according to the production amount in China (Chen et al., 2003). However, with overfishing, high-density aquaculture, and disease infestation, the *L. crocea* industry is encountering serious obstacles for sustainable development. A common parasitic disease caused by *Cryptocaryon irritans* has resulted in high mortalities and great economic losses (Zuo et al., 2012; Buchmann, 2015). It’s essential to analyze the pathogenesis of *C. irritans*. Based on a high-quality sequencing and assembly of *L. crocea* genome, we have constructed a high-density genetic linkage map and revealed temporal gene expression patterns in skin of *L. crocea* in response to *C. irritans* infection (Chen et al., 2019; Kong et al., 2019; Bai et al., 2020). However, the reported studies of *L. crocea* infected with *C. irritans* mainly focused on protein-coding transcripts or peptides on different tissues (Wang et al., 2016; Zheng et al., 2018; Bai et al., 2020). The regulation of immune functions of lncRNAs has not been conducted in *L. crocea* infected with *C. irritans*. This study focuses on the catalog of lncRNA and their immune regulatory functions in *L. crocea*. This work revealed an overall prospect of lncRNAs to improve the present annotation of the genome of *L. crocea* and also provide new insights on the response mechanisms to *C. irritans* infection of fish.

## Materials and Methods

### Data Collection

First, 81 RNA-seq datasets of *L. crocea* were downloaded from the NCBI database^3^. The transcriptome sequencing datasets varied in sexes, ages, tissues, and stress challenges such as cold, hypoxia, and different infections (Supplementary Table S1).

In addition, 20 RNA-seq datasets from skin samples of *C. irritans* infected *L. crocea* performed by our previous work were added into the dataset (Bai et al., 2020). Briefly, 448 healthy *L. crocea* (weight: 25.98 ± 32.72 g, body length 12.32 ± 6.18 cm) were obtained from Fufa Aquatic Products Co., Ltd. (Ningde, Fujian) to carry out a *C. irritans* challenge experiment. Before the challenge experiment, *L. crocea* were acclimated for 15 days in a cement tank (26 ± 0.2°C). A set of 20 healthy fish were randomly selected as the control group. The samples were collected at the beginning of the experiment, and four dying fish were sacrificed at each of the four time points: 24, 48, 72, and 96 h post infection. The skin tissue samples of the fish (16 infected fish and 4 control) were collected, and the RNA in the skin tissue was extracted and sequenced.

### Transcription Assembly and Identification of lncRNA

All the raw data were first trimmed by Trimmomatic software (v0.38) (Bolger et al., 2014) to eliminate the low-quality data reads and adaptor sequences. Then, HISAT2 (v2.1.0) (Kim et al., 2015) was used to align the sequencing reads independently to a reference genome of *L. crocea* (Chen et al., 2019). The alignment results were assembled into transcripts by Cufflinks (v2.1.1) without reference. With orthoDB (v10), BUSCO (v5) was conducted to explore completeness according to conserved ortholog content and evaluate the accuracy of assembly (Simao et al., 2015).

A strict step-wise pipeline was performed to identify lncRNAs from transcripts. First, the transcripts longer than 200 nt were kept for further analysis. Next, three different softwares, CPC2, CNCI, and PLEK, were used to estimate the coding potential of transcripts. CPC2 was based on biologically meaningful sequence features (Kang et al., 2017); CNCI (v2) worked by profiling adjoining nucleotide triplets (Sun et al., 2013); PLEK (v1.2) used a computational pipeline based on an improved k-mer scheme and a support vector machine (SVM) algorithm (Li et al., 2014). Transcripts identified as coding RNAs by any of the three software were removed. Third, the remaining transcripts were searched against the annotation of *L. crocea* based on Swiss-Prot database and Nr database to remove known protein-coding RNAs and small non-coding RNAs classes like mRNA, tRNA, rRNA, miRNA, and snRNA (Chen et al., 2019). The survived transcripts were aligned to four open databases including Pfam^4^, Rfam^5^, Uniprot^6^, and miRBase^7^ using the program pfamscan (Madeira et al., 2019), Infernal (Nawrocki and Eddy, 2013), and BLAST^8^. All transcripts with alignment *E*-value < 1e-6 were removed. Last, the open reading frames (ORFs) of the remaining transcripts were predicted by using ORFfinder^9^. Any transcripts containing ORF more than 100 amino acid were filtered out. The set of remaining transcripts were considered as candidate lncRNAs in this study and used for the further analysis.

### Classification of lncRNAs

lncRNAs were divided into four classes, including lincRNA, exonic lncRNA, intronic lncRNA, and overlapping lncRNA, by using the class code generated from Cuffcompare (Al-Tobasei et al., 2016). The classification was based on the position relationship between lncRNAs and protein-coding genes. lincRNAs, also known as intergenic lncRNAs that did not intersect with any protein-coding genes, were represented as class codes “u” and “p.” Exonic lncRNAs intersected with at least a protein-coding exon were represented as “j,” “e,” “o,” and “x.” Intronic lncRNAs that are not sharing any sequences with exons but exist in introns were represented as “i” or “=.” Overlapping lncRNAs whose introns contain a protein-coding gene were represented as “c.”

### Analysis of Conservation

BLAST was used to evaluate conservation of lncRNAs in *L. crocea* with zebrafish and rainbow trout with an *E*-value < 1e-6 cut off. The data of zebrafish was downloaded from NOCODE database, and data of rainbow trout was retrieved from a previous lncRNAs profiling study (Al-Tobasei et al., 2016; Fang et al., 2018).

### Expression Analysis of lncRNA

To investigate the expression pattern of *L. crocea* under the infection of *C. irritans*, Cuffdiff was used to identify differentially expressed lncRNAs (DElncRNAs) and differentially expressed protein-coding genes (DEgenes). Expression value of lncRNAs and protein-coding genes in each time point group (24, 48, 72, and 96 h post infection) and control group were determined in terms of FPKM. Transcripts expressing differently between any two groups and fulfilling with statistical significance criteria (|log_2_[foldchange]| ≥ 2 and *p*-value < 0.05) were regarded as DElncRNAs and DEgenes.

### Target Gene Prediction of lncRNAs and Gene Clustering

The Pearson’s correlation coefficients (*r*) between each pair of DElncRNAs and DEgenes in *L. crocea* genome were calculated via in-house R scripts (v3.5.3). The DEgene with |*r*| > 0.99 and *p*-value < 0.05 was considered as the target gene of the paired DElncRNA.

Time course sequencing data analysis (TCseq) (v1.6.0) was used to cluster the genes having similar expression patterns. To identify the hub genes, all target genes were subjected to protein-protein interaction (PPI) network analysis with the Cytoscape software (v3.7.2). Three species, *Oryzias latipes*, *Danio rerio*, and *Gasterosteus aculeatus*, were selected as references in the STRING database (Szklarczyk et al., 2017). Genes with degree value (number of genes interacting with a specific gene) ranking top 10% were considered as the hub genes.

### Functional Annotation and Co-expression Network Analysis

All the target genes were searched against the InnateDB^10^ to identify immune-related genes that play important roles in response to the infection of *C. irritans*. GO enrichment and KEGG pathway analysis of the target genes were conducted using OmicShare tools^11^ to get a better understanding of potential functional roles.

The 500 nt sequences located upstream of DElncRNAs and target genes were retrieved from the *L. crocea* genome to identify transcription factor binding sites (TFBS). The retrieved sequences were searched against the AnimalTFDB (v3.0)^12^. The sequences with *p*-value < 1e-6 were identified as potential TFBS.

Cytoscape software was applied to visualize an integrated network between DElncRNAs and target genes based on Pearson’s correlation coefficients and degree values from PPI analysis.

### Quantitative Real-Time PCR (qRT-PCR) Verification

Using a SYBR Premix Ex Taq kit (Invitrogen), qRT-PCR was performed to validate the results of RNA-seq. Primer sets were designed on an online website^13^ (Supplementary Table S2). The thermal cycling profile consisted of 95°C for 5 min, followed by 40 cycles of 95°C for 15 s, and 60°C for 30 s. All the reactions were conducted in technical triplicates and using β-actin as an internal control. The expression levels of genes were analyzed using the comparative threshold (CT) cycle method (Livak and Schmittgen, 2001). Statistical analysis was conducted through one-way analysis of variance (ANOVA).

## Results

### Genome-Wide Identification of lncRNAs in *L. crocea*

**FIGURE 1:**
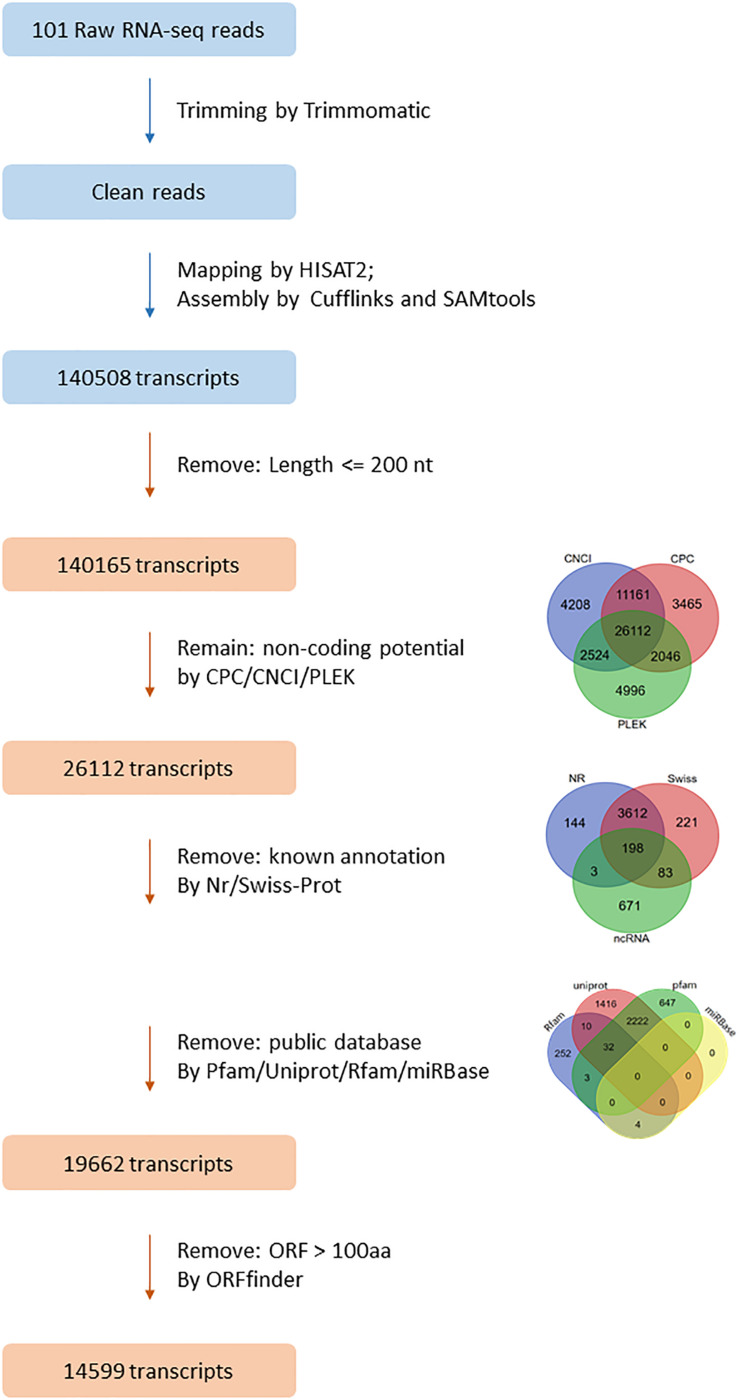
The filtering pipeline for identification of lncRNAs in *L. crocea*. Venn diagrams show the results of three coding potential prediction tools, blast against known annotation, and blast against public database. In the end, a total of 14,599 transcripts were identified as lncRNAs in *L. crocea*.

A total of 1034.7 Gb RNA-seq data was mapped to the *L. crocea* genome by using HISAT2. The average mapping ratio for samples was 80.41% (Supplementary Table S3). The mapped reads were assembled into 140,508 transcripts by using Cufflinks. A set of 14,599 lncRNAs from 13,673 loci of the assembled transcripts were identified (Figure 1). BUSCO was used to evaluate the accuracy of assembly, and 78.6% complete single-copy or duplicated orthologs were identified (Supplementary Figure S1).

**FIGURE 2:**
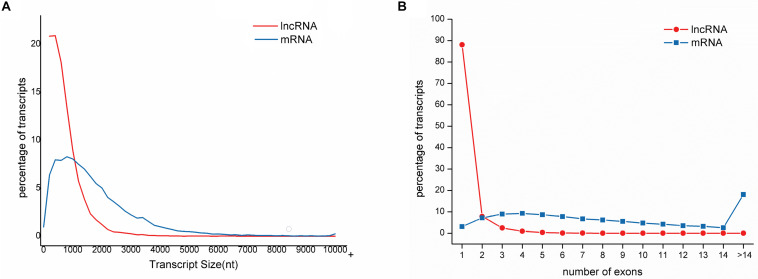
Distribution of sequence length and exon number of lncRNAs compared to mRNAs in large yellow croaker. **(A)** lncRNAs is much shorter than mRNA. The majority of lncRNAs (73.27%) is shorter than 1000 nt while only 31.45% for mRNAs. **(B)** lncRNAs have less exons than mRNAs. Of lncRNAs, 88.06% have only one exon while 18.06% of mRNAs have exons more than 14.

The length of lncRNAs ranged from 201 nt to 10,080 nt, with an average sequence length of 684 nt. The lengths of N50, N70, and N90 transcripts were 999 nt, 726 nt, and 444 nt, respectively (Table 1). The number of exons in each lncRNA ranged from 1 to 14, with an average of 1.19 exon. The majority of lncRNAs (88.06%) contained only a single exon. In contrast, the average length of mRNA was 1485, and the average number of exons in mRNA was 9.45 ranging from 1 to 143. Compared to mRNAs, lncRNAs have fewer exons and shorter transcript length, which is in accordance with the previous study (Table 2 and Figure 2) (Al-Tobasei et al., 2016; Bakhtiarizadeh and Salami, 2019).

**TABLE 1:** Summary statistic for lncRNA dataset identified in this study.

Items	Statistics value
Total transcripts	14599
Total loci	13673
N50 length (nt)	999
N60 length (nt)	857
N70 length (nt)	726
N80 length (nt)	596
N90 length (nt)	444
Total length (nt)	12065150
Max length (nt)	10080
Min length (nt)	201
Average length (nt)	684
Number of exons	
=1	12858 (88.06%)
≤2	14009 (95.95%)
≤3	14374 (98.45%)

**TABLE 2:** Different characteristics of lncRNAs and mRNAs identified in this study.

Items	lncRNA	mRNA
Total transcripts	14599	23807
Average length (nt)	684	1485
Average number of exons	1.19	9.45
Largest number of exons	14	143

To explore the conservation of lncRNAs across different species, the 14,599 identified lncRNAs in *L. crocea* were blasted with lncRNAs of zebrafish and rainbow trout. Only 18 (0.12%) and 75 (0.51%) conserved lncRNAs were found in zebrafish and rainbow trout, respectively. The weak similarity proved the low level of conservation of lncRNAs between species.

Based on the position relationship with protein-coding genes, lncRNAs were divided into four classes, including lincRNA, exonic lncRNA, intronic lncRNA, and overlapping lncRNA. Among the 14,599 lncRNAs in our study, 10,008 (68.55%) lincRNAs, 1774 (12.15%) exonic lncRNAs, 2808 (19.23%) intronic lncRNAs, and 10 (0.07%) overlapping lncRNAs were identified. From the result, we can conclude that the majority of lncRNAs are lincRNAs that do not share any part with protein-coding genes.

### The DElncRNAs and Target Genes Under the Infection of *Cryptocaryon irritans*

Using the transcript file produced in the previous step and the aligned reads from 20 RNA-seq datasets under the infection of *Cryptocaryon irritans*, 77 DElncRNAs were identified by Cuffdiff with the threshold of |log_2_FC| ≥ 2, *p*-value < 0.05. The 77 DElncRNAs were clustered into seven categories. Cluster1, cluster2, cluster3, cluster5, and cluster6 include lncRNAs whose expression peaks at 0, 24, 48, 72, and 96 h post-infection (Supplementary Figure S2). Cluster4 and cluster7 contain lncRNAs with continuous increase and decrease expression patterns, respectively.

Comparing the position of DElncRNAs and genes, 15 DElncRNAs were identified to locate in the genome of 46 genes of *L. crocea*. All genes were annotated by Swiss-Prot and 11 of them were related to immune system including C1QL4, CCL28, TNNT3, ANGPT1, BCAP29, CASQ1, S100A13, TNNI2, SERINC3, PDE1B, and STX2.

To identify target genes for DElncRNAs, we calculated the Pearson’s correlation coefficients between 77 DElncRNAs and 1,852 differential expressed genes (Supplementary Table S4). A total of 1,204 pairs with high correlation coefficient (|*r*| > 0.99, *p*-value < 0.05) including 60 DElncRNAs and 567 differential expressed genes were identified. The 567 genes were considered as the target genes in this study. Among the 1,204 high-confidence pairs, most (94.27%) were positive correlation. The number of target genes for each lncRNA ranged from 1 to 98 (TCONS_00126625 was the lncRNA that associated with the most genes), and the average number is 20. About 70% of lncRNAs have fewer than 18 target genes.

### GO and KEGG Enrichment Analysis of Target Genes

All the target genes were annotated by Swiss-Prot database. The target genes were searched against the InnateDB database, which including genes involved in the innate immune response. A total of 71 genes were identified as immune-related genes, such as BCL2L1, IL17C, IL1R2, and MMP9 (Supplementary Table S5).

**FIGURE 3:**
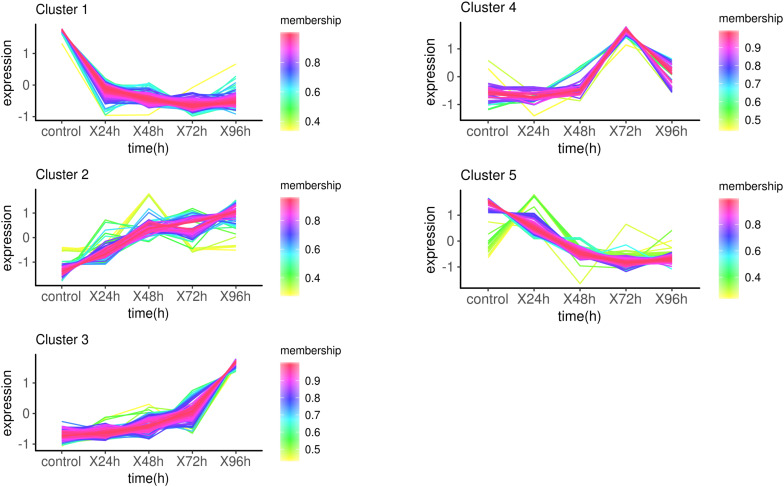
Expression patterns of target genes in infection analysis. Membership values indicated the degree that a transcript belonged to this cluster. The more degree, the more relevance.

The target genes were clustered into five groups according to the trend of expression pattern (Figure 3). GO enrichment and KEGG pathway analysis were performed for each cluster. With the threshold of *p*-value < 0.01, a total of 252 GO terms were enriched in 5 clusters (Supplementary Table S6 and Supplementary Figure S3). Several GO terms related to phosphorylation and metabolic process were enriched in cluster1. Ion channel activity and transport activity were significantly enriched in cluster2, which was continually up-regulated within 96 h. Remarkably, a lot of GO terms enriched in cluster3 are related to diverse aspects of innate immune response, including toll-like receptor, cytokine, signaling pathway, and defense response. This was attributed to the up-expression of several immune-related genes, including Tlr5, MMP9, CD2AP, IL1R2, and TNFAIP3. Go terms involved in response to food were highly enriched in cluster 4 as a result of CARTPT, which encodes a preproprotein that plays a role in appetite and energy balance. The up-regulation of CARTPT in 72 h may stimulate the appetite and make response to food. The infection of *C. irritans* leading to the depression of movement, which may cause the enrichment of GO terms related to sarcoplasm in cluster5. The GO terms relevant to the metabolic process and respiratory chain were also enriched significantly in cluster5, which represented the down-expressed biological process after the infection of *C. irritans*.

With a cut-off of *p*-value < 0.05, there were 9 (cluster1), 7 (cluster2), 17 (cluster3), 9 (cluster4), and 33 (cluster5) KEGG pathway enriched in five groups respectively (Supplementary Table S7). Bacterial invasion of epithelial cells (ko05100) and *Staphylococcus aureus* infection (ko05150) were enriched in cluster1, which explained cell response to infection in the beginning. Complement and coagulation cascades (ko04610) and TGF-beta signaling pathway (ko04350) were only significantly enriched in cluster2. Cytokine-cytokine receptor interaction (ko04060) was enriched both in cluster2 and cluster3. There are several disease-related pathways that were significantly enriched in cluster3, such as amoebiasis (ko05146), transcriptional misregulation in cancers (ko05202), microRNAs in cancer (ko05206), and transcription misregulation in cancer (Supplementary Figure S4). Regulation of lipolysis in adipocyte (ko04923) and biosynthesis of amino acids (ko01230) were enriched in cluster4. Some metabolic pathways, like metabolic pathways (ko01100) and fatty acid metabolism (ko01212), were significantly enriched in cluster5.

### Hub Genes and Interaction Networks Between DElncRNAs and Target Genes

A total of 466 target genes were annotated by using STRING database to predict interactions between proteins. PPI analysis was performed for each of the target clusters (Supplementary Figure S5). With the help of the PPI network, we can calculate the interaction of genes by degree method in the same cluster. Finally, a total of 21 genes ranked in the top 10% were selected as the hub genes (Table 3). The number of genes interacting with hub genes ranged from 22 (cluster4) to 58 (cluster1). Syne2 was identified as the top hub gene in cluster1. Notably, nine hub genes identified in the up-regulated cluster contained six genes related to cancer, immune cell, and immune response, including Bcl3, Rhog of cluster2, and CD2AP, RAB25, Tlr5, ZBTB7B of cluster3. Pbk involved in the activation of lymphoid cells was the only hub gene indicated in cluster4. In the hub genes of cluster5, Gp5 can encode protein that is essential for viral DNA ejection into the host cell, which may cause early viral infection in fish.

**TABLE 3:** The hub genes of each cluster.

Cluster	Rank	Gene name	Score	Annotation (Uniprot)
1	1	Syne2	58	Nesprin-2
1	2	PODN	54	Podocan
1	2	LRRC20	54	Leucine-rich repeat-containing protein 20
1	4	RHOBTB2	52	Rho-related BTB domain-containing protein 2
1	5	Myh8	50	Myosin-8
1	6	ANK2	48	Ankyrin-2
2	1	Bcl3	44	B-cell lymphoma 3 protein
2	2	RND1	38	Rho-related GTP-binding protein Rho6
2	3	Rhog	36	Rho-related GTP-binding protein RhoG
2	4	zgc:110179	34	Ras-like protein family member 11A-like protein
3	1	CD2AP	46	CD2-associated protein
3	2	RAB25	34	Ras-related protein Rab-25
3	2	Tlr5	34	Toll-like receptor 5
3	4	ZBTB7B	32	Zinc finger and BTB domain-containing protein 7B
3	4	KLF5	32	Krueppel-like factor 5
4	1	pbk	22	Lymphokine-activated killer T-cell-originated protein kinase
5	1	GP1BB	46	Platelet glycoprotein Ib beta chain
5	1	Gp5	46	Baseplate central spike complex protein gp5
5	3	PKM	44	Pyruvate kinase PKM
5	3	TESK1	44	Dual specificity testis-specific protein kinase 1
5	5	ASB5	36	Ankyrin repeat and SOCS box protein 5
5	5	spata5l1	36	Spermatogenesis-associated protein 5-like protein 1
5	5	atp2a1	36	Sarcoplasmic/endoplasmic reticulum calcium ATPase 1

An integrated lncRNA-target genes interaction network included PPI network and co-expressed lncRNA-target genes was merged, which comprised 59 lncRNAs and 466 target genes, including 8057 interactions (Supplementary Figure S6). Except for TCONS_00002999 and PPL, all DElncRNAs and target genes merged as a complex interaction network.

### Transcription Factor Binding Sites of DElncRNAs and Target Genes

It is reported that genes with strongly correlated mRNA expression profiles are more likely to have their promoter regions bound by a common transcription factor (Allocco et al., 2004). To predict TFBS in the promotor regions of DElncRNAs and target genes, 500 nts upstream of sequences were scanned. According to AnimalTFDB database, the number of potential TFBS for all DElncRNAs and target genes was 294 and 595, respectively. There were 282 transcription factors that may both bind to DElncRNAs and target genes. Indeed, among the 1,204 high-confidence pairs of DElncRNAs and target genes, 504 pairs had at least one common transcription factor, and the average number was 3.20. Over 50% (264/504) of these pairs had at least one common immune-related transcription factor. The result may infer that the co-expressed genes were likely controlled by similar regulatory mechanisms and were regulated by the same transcription factors.

### Validation of DElncRNAs Using Quantitative Real-Time PCR

To validate the transcriptional expression, 4 DElncRNAs (TCONS_00012911, TCONS_00105595, TCONS_00047797, TCONS_00068543) were selected to perform real-time PCR in this study. The qRT-PCR results correlated well with the results obtained through RNA-seq (Supplementary Figure S7).

## Discussion

### Identification of lncRNAs in *L. crocea*

The ENCODE project showed that only 1–2% of the human genome encode for protein and the numerous non-coding RNAs include tRNAs, rRNAs, micro RNAs, lncRNAs, and other non-coding RNAs. With the development of RNA sequencing technologies, improved epigenomic techniques, and computational prediction methods, an increased focus was motivated to understand the roles of non-coding RNAs in biology. As an indispensable part of the genome, lncRNAs engage in diverse biological processes across every branch of life and required more analyses (Quinn and Chang, 2016). However, low conservation levels between taxa make the functional annotation difficult and block further understanding. A comparison of the lincRNAs in zebrafish, human, and mouse revealed that only 29 lincRNAs were conserved between fish and mammals (Hezroni et al., 2015). Therefore, further works call for a unique catalog of lncRNAs based on a large number of datasets for specific fish. Analysis of lncRNAs has been performed on other species in teleost. Al-Tobasei et al. (2016) analyzed more than two billion sequencing reads from four independent datasets and identified 31,195 lncRNAs of rainbow trout. Li et al. (2018) collected 103 RNAseq datasets to identify 72,276 high-confidence lncRNAs in tilapia. As an important economic aquaculture fish, no systematic analysis of lncRNAs has been conducted for *L. crocea*. In this study, a total of 14,599 lncRNAs were identified from 101 RNAseq datasets with a strict pipeline and divided into four classes based on the position relationship with protein-coding genes. In accordance with the previous study, lncRNAs identified in this study have some special characters compared to mRNAs like fewer exons, shorter transcript lengths, and low conversation levels (Al-Tobasei et al., 2016; Bakhtiarizadeh and Salami, 2019).

### lncRNAs Play Important Roles in the Immune Response

Innate immune reactions contribute to the fundamental defense strategy of fish in response to various infection agents. It’s crucial to recognize the danger and activate subsequent signaling cascades. Previous studies have demonstrated that lncRNAs have a profound impact on the immune response in teleost (Jiang et al., 2016; Valenzuela-Munoz et al., 2018). We focused on the infection analysis of *C. irritans* and identified a lot of DElncRNAs with immune-related target genes. One of the important discoveries was the identification of TLRs families.

**FIGURE 4:**
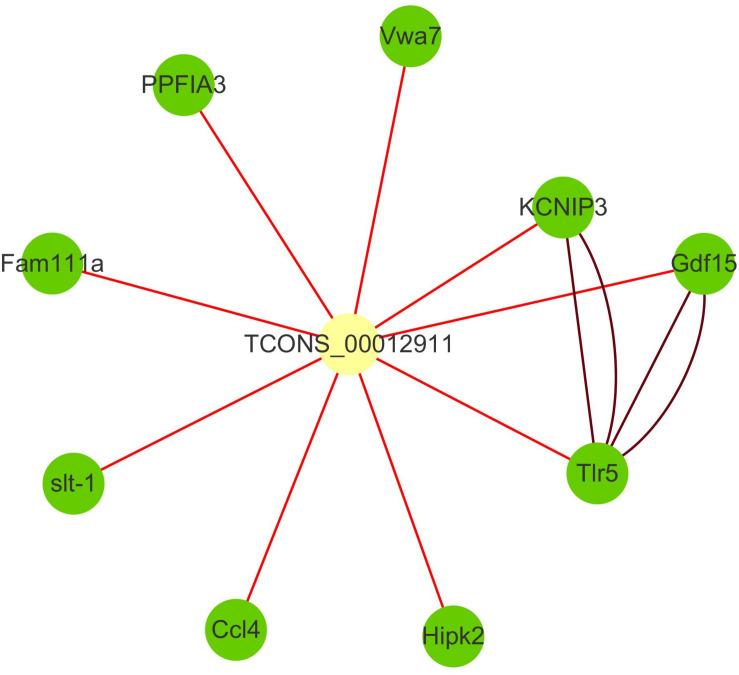
Interaction network of lncRNA TCONS_00012911 with putative target genes. Yellow square nodes: lncRNA TCONS_00012911; green nodes: target genes; brown lines: interactions between genes based on STRING database; red lines: positive interactions between lncRNAs and genes (Pearson’s correlation coefficient > 0.99).

The family of toll-like receptors (TLRs) have been regarded as key partners in recognition of specific pathogen-associated molecular patterns (PAMPs) and activating signal pathways related to inflammatory response. TLR signaling through PAMPs recognition divided into myeloid differentiation primary response protein 88 (MyD88)-dependent and -independent signaling pathway (Li et al., 2012). Numerous studies revealed that fish TLRs have high structural similarity to the mammalian TLR system and might also be similar in the regulation of immune response (Rebl et al., 2010; Palti, 2011). In recent years, 20 TLRs, including a group of fish-specific TLRs, were identified (Zhang et al., 2013). And several studies have proved the immune-related roles of TLRs under infectious stimulation in teleost including zebrafish, fugu (Meijer et al., 2004), and orange-spotted grouper (*Epinephelus coioides*) (Li et al., 2012). As a significant member of TLRs, TLR5 has been shown to recognize the flagellin protein component of bacterial flagella and be responsible for flagellin-mediated NF-κB activation (Hayashi et al., 2001). Previous works also identified TLR5 in bony fish, such as zebrafish, fugu (Meijer et al., 2004), catfish (*Pangasianodon hypophthalmus*) (Schmitz et al., 2017), Japanese flounder (*Paralichthys olivaceus*) (Hwang et al., 2010), and rainbow trout (*Onchorhynchus mikiss*) (Tsujita et al., 2004). In this study, toll-like receptor 5 signaling pathway and MyD88-dependent toll-like receptor signaling pathway were significantly enriched in up-regulated cluster, and TLR5 is the hub genes of cluster3. With the remote position and high correlation, we speculated that lncRNA TCONS_00012911 might positively regulate TLR5 through trans way (Figure 4). Indeed, they had seven common transcription factors binding sites including five immune-related ones in the promoter region. In addition, as an important part of Toll/interleukin-1 (Il-1) receptor domain (TIR domain), IL1R2 was also indicated in cluster3. It’s well known that TIR domain was a protein–protein interaction domain that occurs in a large group of host defense associated proteins from diverse species (Meijer et al., 2004). Therefore, we speculated that the expressions of TLR5 and IL1R2 transcripts were increased under the infection of *C. irritans* and might be crucial for the innate immune response.

Besides TLR families, other immune-related genes were identified in target genes. In cluster3, which can represent the up-regulated genes, the other immune-related enriched GO terms can be divided into three types, including the cytokine secretion (e.g., GO:0050663, GO:0050707, GO:0001819), the innate immune response (e.g., GO:0002218, GO:0002758, GO:0045088), and the defense response (e.g., GO:0031349, GO:0042742, GO:0031347). KEGG pathway also discovered several immune-related pathways, including ECM-receptor interaction (ko04512) and microRNAs in cancer (ko05206) in cluster3. The ECM functions as ligands for cell surface receptors such as integrins, dystroglycans, toll-like receptors (TLRs), and regulate cellular signaling and immune cell dynamics (Bhattacharjee et al., 2019). MicroRNAs in cancer pathway are not only indicating the function lncRNAs played in immune response but also emphasizing the interaction between microRNAs and lncRNAs. A lot of immune-related genes are involved in the enriched GO terms and pathways, including TLR5, CD2AP, and MMP9. CD2 associated protein (CD2AP) is an adaptor protein that couples endocytic proteins to the actin cytoskeleton. It can improve the protective antibody response in viral infection by tuning TCR signaling (Raju et al., 2018). Members of the matrix metalloproteinase (MMP) family are essential for the remodeling of the extracellular matrix in a number of biological processes, including a variety of immune responses. MMP9 is highly expressed in specific myeloid cell populations in which it plays a role in the innate immune response and was identified in zebrafish (Yoong et al., 2007).

In cluster2, genes that showed a continually increased pattern were collected. GO enrichment analysis showed that ion channel activity terms (e.g., GO:0005247, GO:0008308, GO:0005254) were significantly enriched. Ion channels and transporters help to establish and control cell hydrophobic lipid membranes. Analysis has revealed important roles of ionic signals in lymphocyte development and innate and adaptive immune responses (Feske et al., 2015). KEGG pathway analysis showed that the complement and coagulation cascades pathway (ko04610), cytokine-cytokine receptor interaction pathway (ko04060), and disease-related pathways (ko05020, ko05322) were significantly enriched in cluster2. Coagulation and complement are two distinct systems with unique pathophysiological roles, and both serve as innate defense against external threats (Oikonomopoulou et al., 2012). Cytokine-cytokine receptor interaction pathway plays important roles in immune response and has been identified in several previous studies of teleost under bacterial or virus infection (Peng et al., 2019; Zhang et al., 2019). In cluster1, genes highly expressed in 0 h were collected. GO enrichment showed terms related to ATP generation (e.g., GO:0006757, GO:0046031) and nucleoside diphosphate metabolic process (e.g., GO:0009135, GO:0009179, GO:0006165) were enriched. It’s well known that ATP is essential to energy support, and the decrease of ATP may be related to poor energy generation and induce the mortalities of fish in the experiment group. These results suggest that lncRNAs regulate the targeted genes and play important roles in immune response to the infection of *C. irritans* in *L. crocea*.

### The Transcriptional Regulation Roles of lncRNAs in Target Genes

It’s well known that lncRNAs can regulate genes in *cis* or *trans* ways, but the mechanism underlying the transcriptional regulation has not been understood (Kopp and Mendell, 2018). Previous studies suggested that the co-expressed genes were likely regulated by the same transcription factors (Allocco et al., 2004; You et al., 2019). In this work, over 40% of high-confidence pairs had at least one common transcription factor. The similarity of DElncRNAs and target genes in transcription factor may suggest a potential function of lncRNAs to regulate target genes. For example, according to the remote position and similar expression patterns, TCONS_00040064 might positively regulate MMP9 through trans way in this study. And they have four common transcriptional factor binding sites in the promoter region, which may explain the regulation of TCONS_00040064 for MMP9.

The regulation and expression of genes are intricate and are affected by complicated factors. In this study, an integrated network comprised of 59 lncRNAs and 481 target genes, including 8057 interactions, was merged and visualized the complicated relationship between lncRNAs and protein-coding genes that differentially expressed infected by *C. irritans*. The further studies and experiments about interactions of lncRNA, miRNA, and protein-coding RNA are required to understand the regulatory process in immune and other biological processes.

## Conclusion

To generate a comprehensive transcriptome of *L. crocea*, 101 RNAseq datasets were used, and a total of 14,599 lncRNAs were identified through a strict pipeline. DE analysis identified 77 DElncRNAs in response to the infection of *C. irritans*, and 567 differentially expressed protein-coding genes were regarded as target genes. A set of immune-related GO terms and KEGG pathways were enriched, in which immune genes including TLR5, MMP9, CD2AP, and IL1R2 were up-regulated significantly. This study provides a new insight for lncRNAs annotation in *L. crocea* genome and helps to deepen the understanding of the roles of lncRNAs in innate immune response to *C. irritans*.

## Data Availability Statement

All datasets generated for this study are included in the article/Supplementary Material.

## Author Contributions

PX and WZ conceived and supervised the study. XZ, TZ, and PX designed and managed the experiments, and wrote the manuscript. XZ, BC, HB, and YB performed the analysis and designed the charts and tables. XZ, YB, JZ, FP, YW, LC, YS, QK, and JC conducted the experiments. All authors have read and approved the manuscript.

## Conflict of Interest

QK, WZ, JC, and PX were employed by the State Key Laboratory of Large Yellow Croaker Breeding, which was constructed by the Ningde Fufa Fisheries Company Limited. The remaining authors declare that the research was conducted in the absence of any commercial or financial relationships that could be construed as a potential conflict of interest.

## Footnotes

1 http://www.noncode.org/

2 https://www.gencodegenes.org/

3 https://www.ncbi.nlm.nih.gov/

4 http://pfam.xfam.org/

5 http://rfam.xfam.org/

6 https://www.uniprot.org/

7 http://www.mirbase.org/

8 https://blast.ncbi.nlm.nih.gov/Blast.cgi

9 https://indra.mullins.microbiol.washington.edu/sms2/orf_find.html

10 https://www.innatedb.ca/

11 https://www.omicshare.com/tools/Home?l=en-us

12 http://bioinfo.life.hust.edu.cn/AnimalTFDB/

13 https://www.genscript.com/tools/real-time-pcr-taqman-primer-design-tool

## Supplementary Material

Supplementary Figure 1 The results of BUSCO softwares.

Supplementary Figure 2 Expression patterns of DElncRNAs in infection analysis. Membership values indicated the degree that a transcript belonged to this cluster. The more degree, the more relevance.

Supplementary Figure 3 The top 20 GO terms enriched in each clusters.

Supplementary Figure 4 Significantly differentially expressed genes identified in diverse KEGG pathways in cluster3. Red boxes indicate significantly differential expression and black boxes indicate unchanged expression. **(A)** Cytokine-cytokine receptor interaction pathway. **(B)** ECM-receptor interaction pathway. **(C)** MicroRNAs in cancer pathway. **(D)** Transcription misregulation in cancer pathway.

Supplementary Figure 5 The PPI networks of target genes. Nodes represent target genes. The bigger node and the darker color mean the higher degree value. Line represent interactions between two genes. The wider line and darker color mean the closer interaction.

Supplementary Figure 6 Integrated lncRNA-target genes interaction network constructed based on co-expressed genes and STRING-derived PPI. Yellow square nodes: DElncRNAs; green nodes: target genes; brown lines: interactions between genes; red lines: positive interactions between lncRNAs and genes (Pearson’s correlation coefficient > 0.99); blue lines: negative interactions between lncRNAs and genes (Pearson’s correlation coefficient < −0.99). The Pearson’s coefficient or interaction value (based on STRING database) of each pairwise was shown in Supplementary Tables 4, 8, respectively.

Supplementary Figure 7 Validation of RNA-seq data by qRT-PCR. Orange bar: RT-PCR; green bar: RNA-seq. **(A)** TCONS_00012911. **(B)** TCONS_00105595. **(C)** TCONS_00047797. **(D)** TCONS_00068543.

Supplementary Table 1 Data collection.

Supplementary Table 2 Primer sets for qRT-PCR.

Supplementary Table 3 The mapping ratio for samples.

Supplementary Table 4 Interaction between DElncRNAs and target genes.

Supplementary Table 5 Target genes identified by the InnateDB as immune genes.

Supplementary Table 6 The top 20 significantly enriched GO term of target genes.

Supplementary Table 7 The significantly enriched KEGG pathways of target genes.

Supplementary Table 8 Interaction between target genes.
