# Supplementary figures and images for: Identification and Expression Analysis of Long Non-coding RNA in Large Yellow Croaker (Larimichthys crocea) in Response to Cryptocaryon irritans Infection
Source: Front Genet. 2020 Nov 12;11:590475. doi: 10.3389/fgene.2020.590475 (PMC7689269; doi:10.3389/fgene.2020.590475)

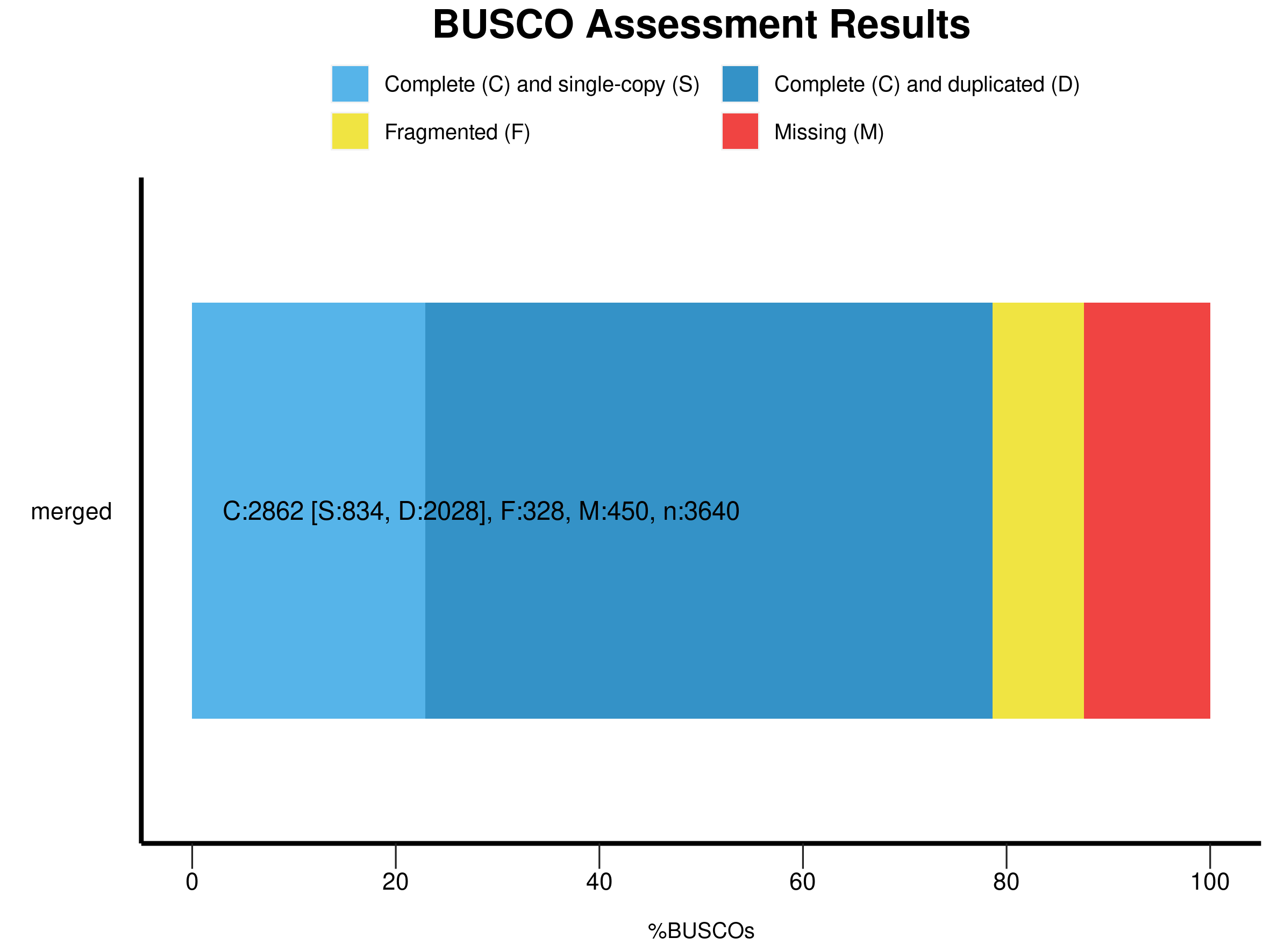

Supplement: Supplementary Figure 1 — The results of BUSCO softwares. [file Image_1.TIFF]

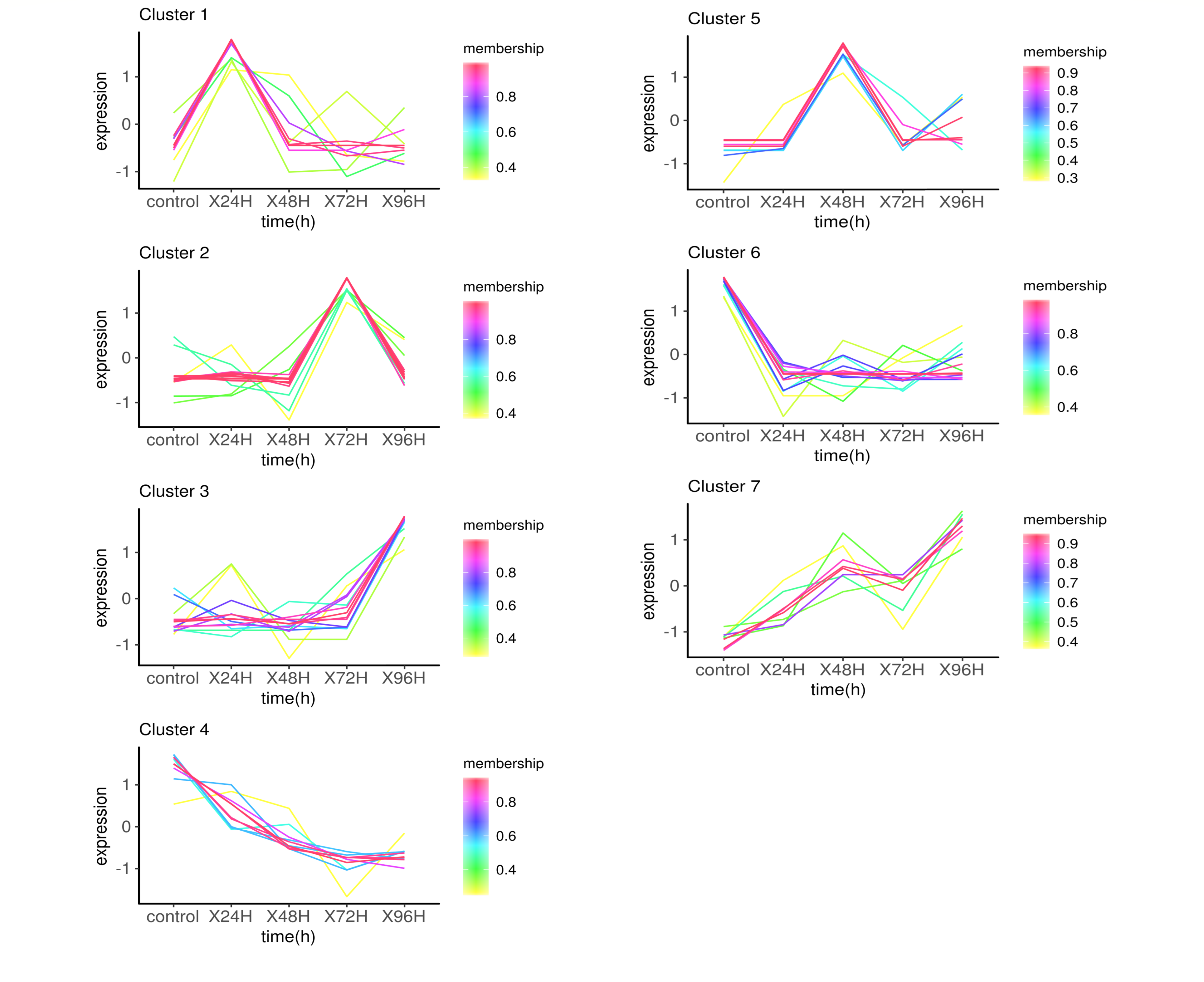

Supplement: Supplementary Figure 2 — Expression patterns of DElncRNAs in infection analysis. Membership values indicated the degree that a transcript belonged to this cluster. The more degree, the more relevance. [file Image_2.TIFF]

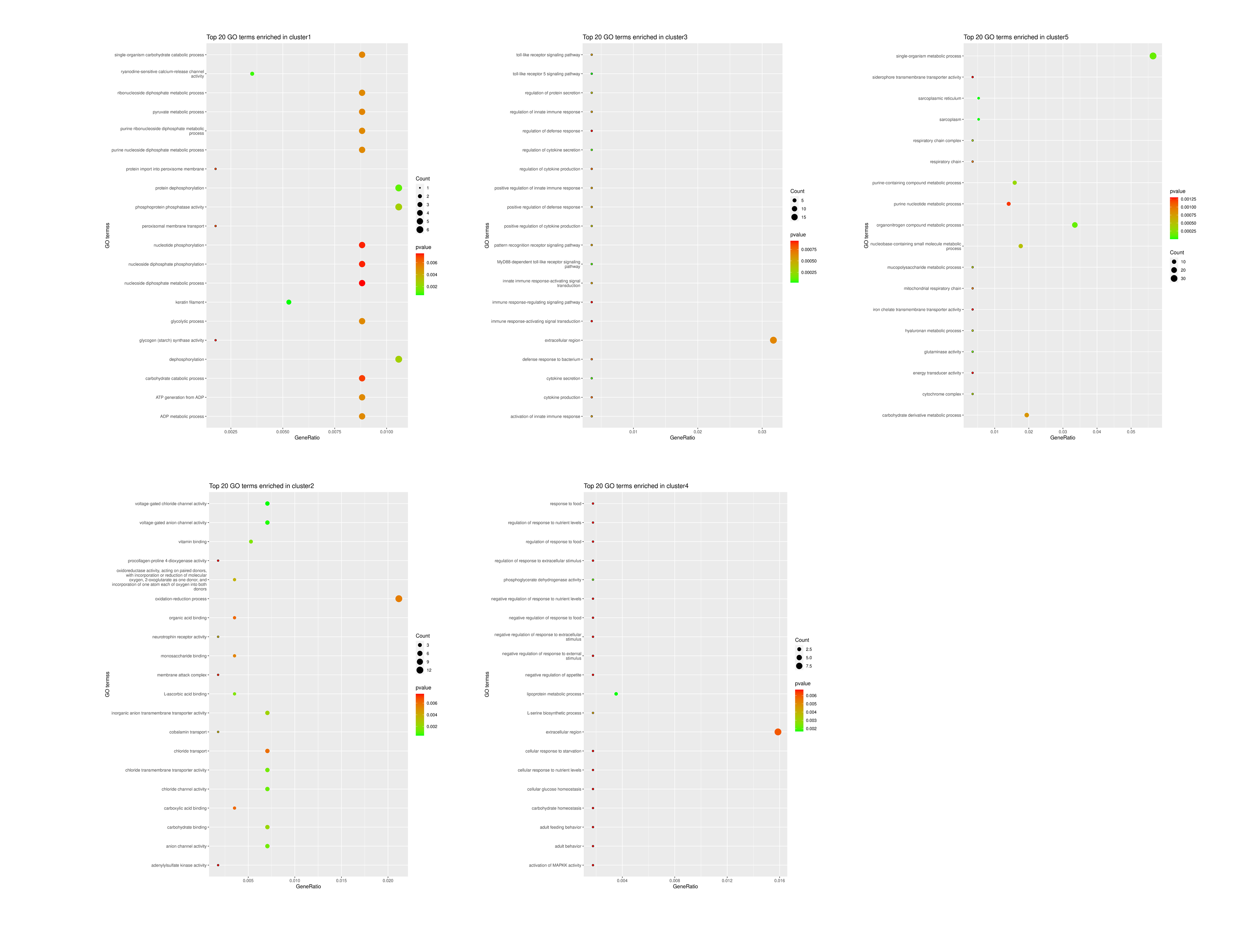

Supplement: Supplementary Figure 3 — The top 20 GO terms enriched in each clusters. [file Image_3.TIF]

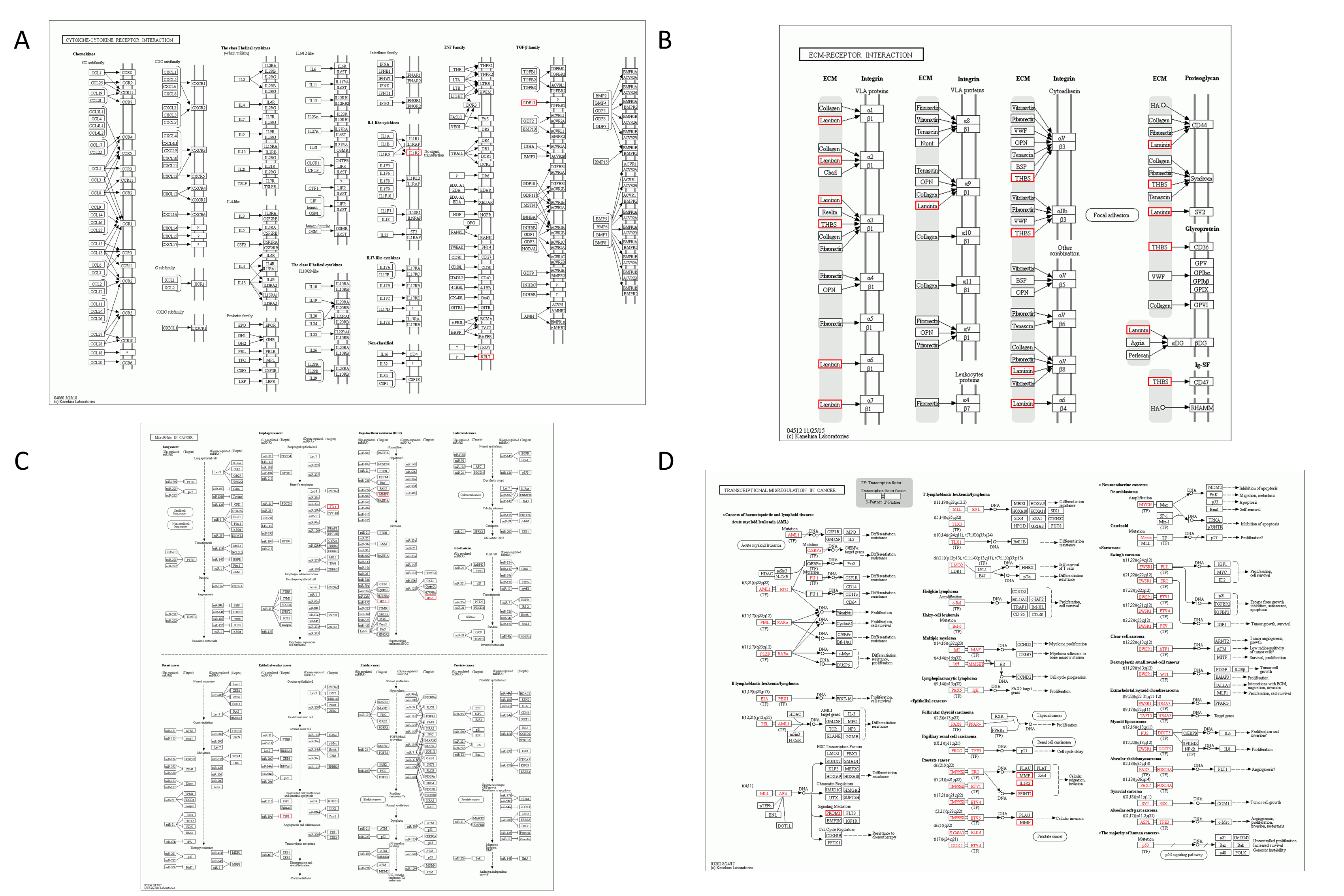

Supplement: Supplementary Figure 4 — Significantly differentially expressed genes identified in diverse KEGG pathways in cluster3. Red boxes indicate significantly differential expression and black boxes indicate unchanged expression. (A) Cytokine-cytokine receptor interaction pathway. (B) ECM-receptor interaction pathway. (C) MicroRNAs in cancer pathway. (D) Transcription misregulation in cancer pathway. [file Image_4.TIF]

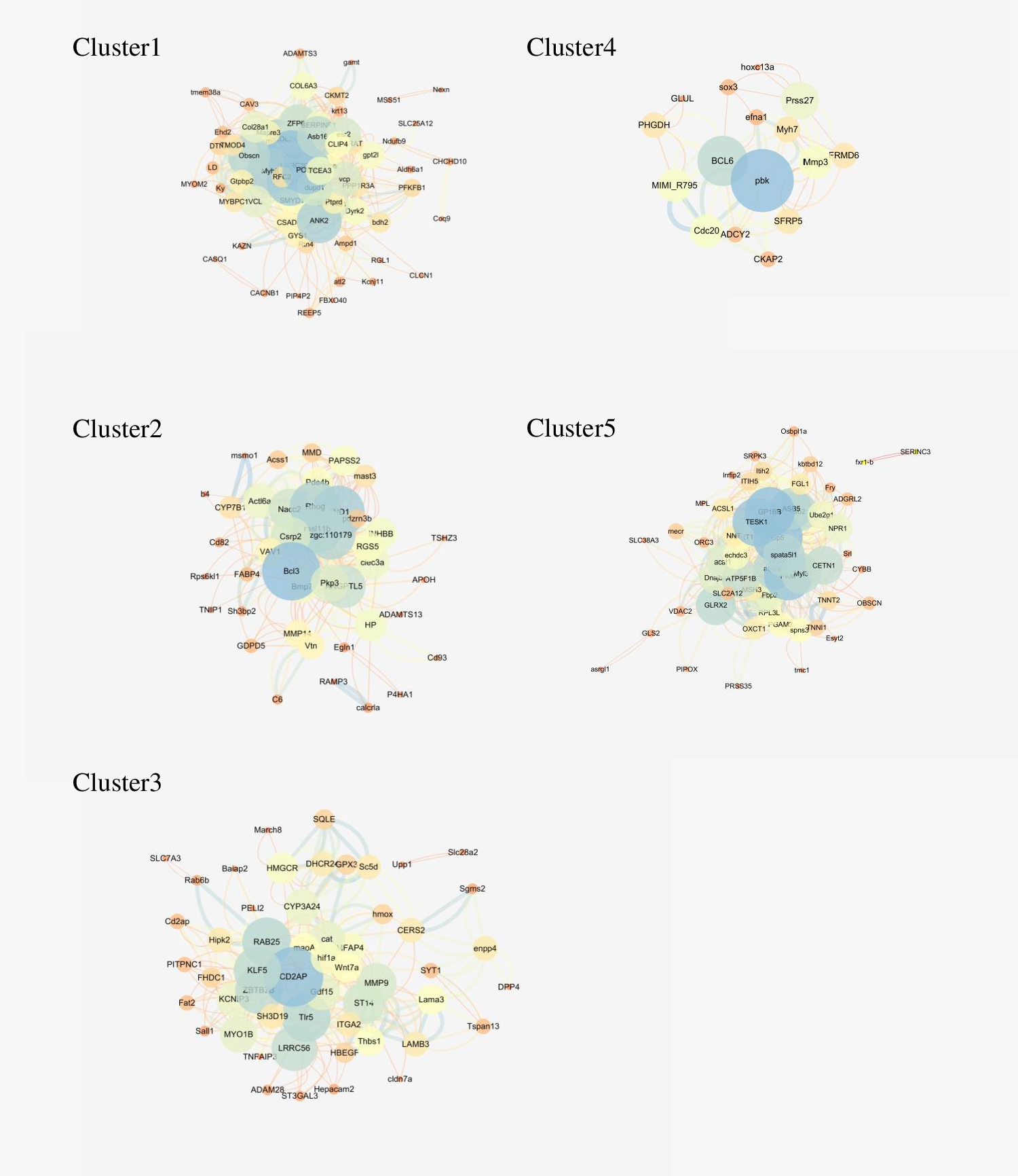

Supplement: Supplementary Figure 5 — The PPI networks of target genes. Nodes represent target genes. The bigger node and the darker color mean the higher degree value. Line represent interactions between two genes. The wider line and darker color mean the closer interaction. [file Image_5.TIFF]

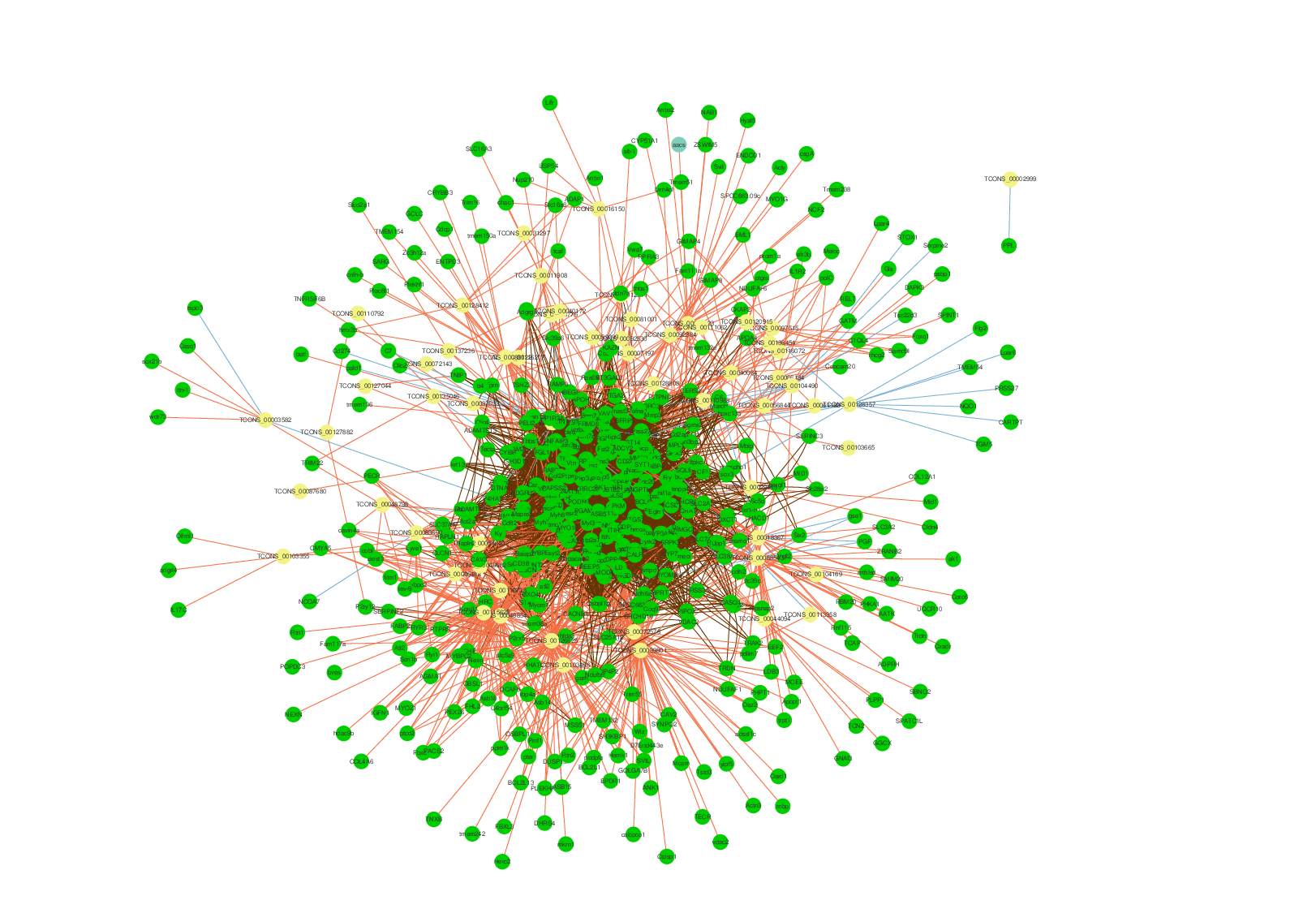

Supplement: Supplementary Figure 6 — Integrated lncRNA-target genes interaction network constructed based on co-expressed genes and STRING-derived PPI. Yellow square nodes: DElncRNAs; green nodes: target genes; brown lines: interactions between genes; red lines: positive interactions between lncRNAs and genes (Pearson’s correlation coefficient > 0.99); blue lines: negative interactions between lncRNAs and genes (Pearson’s correlation coefficient < −0.99). The Pearson’s coefficient or interaction value (based on STRING database) of each pairwise was shown in Supplementary Tables 4, 8, respectively. [file Image_6.TIFF]

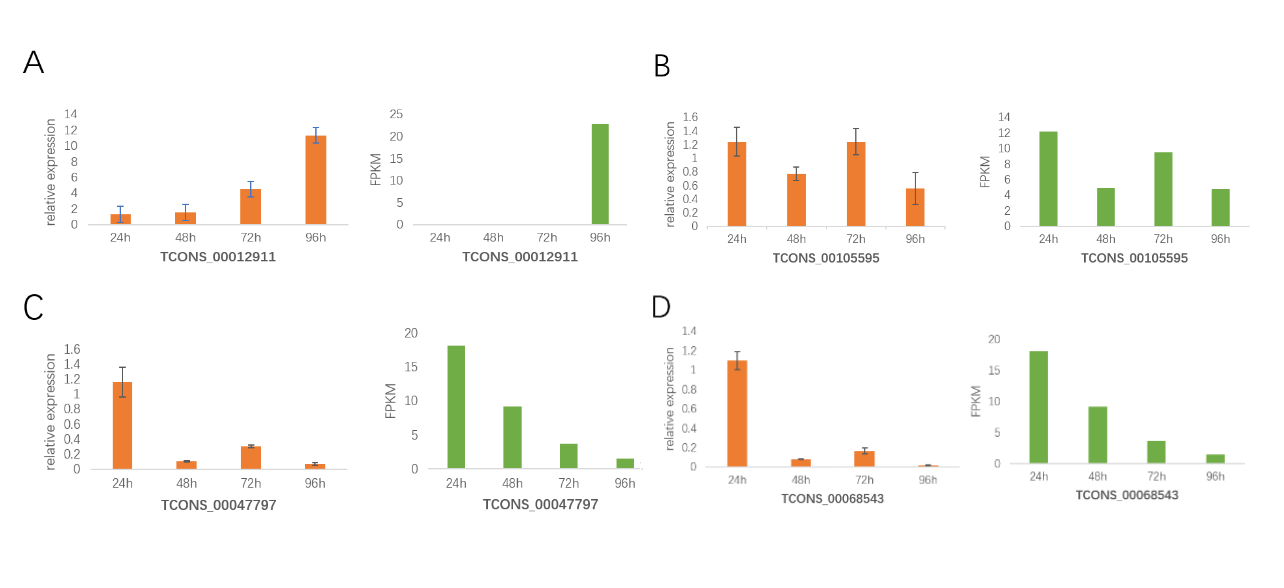

Supplement: Supplementary Figure 7 — Validation of RNA-seq data by qRT-PCR. Orange bar: RT-PCR; green bar: RNA-seq. (A) TCONS_00012911. (B) TCONS_00105595. (C) TCONS_00047797. (D) TCONS_00068543. [file Image_7.TIFF]
